# A hsa_circ_001726 axis regulated by E2F6 contributes to metastasis of hepatocellular carcinoma

**DOI:** 10.1186/s12885-023-11703-7

**Published:** 2024-01-02

**Authors:** Jiaoyu Ai, Wanlin Zhang, Wensheng Deng, Likun Yan, Lidong Zhang, Zongjing Huang, Ziyi Wu, Junhua Ai, Hai Jiang

**Affiliations:** 1 Department of Gastroenterology, The First Affiliated Hospital, Jiangxi Medical College, Nanchang University, Nanchang, Jiangxi China; 2 Department of Clinical Laboratory, The First Affiliated Hospital, Jiangxi Medical College, Nanchang University, Nanchang, Jiangxi China; 3 Department of General Surgery, The First Affiliated Hospital, Jiangxi Medical College, Nanchang University, Nanchang, Jiangxi China

**Keywords:** Hsa_circ_001726, E2F6, PRMT9, Metastasis, Hepatocellular carcinoma

## Abstract

**Background:**

CircRNAs participate in the development of hepatocellular carcinoma (HCC). This work aims to explore the key tumor promoting circRNA as a gene therapy target.

**Methods:**

The differentially expressed gene circRNAs in HCC tumor tissues was identified by mining GSE121714 dataset. EdU staining, wound healing, transwell invasion assay, TUNEL staining and western blotting examined proliferation, migration, invasion, apoptosis and epithelial mesenchymal transition (EMT). Xenograft mouse model and orthotopic transplantation tumor mouse model were constructed to verify the role of hsa_circ_001726 in growth and metastasis of HCC. The relationship among CCT2, E2F6, hsa_circ_001726, miR-671-5p and PRMT9 was identified by RNA-fluorescence in situ hybridization, luciferase reporter assay and RNA Immunoprecipitation.

**Results:**

Eleven differentially expressed circRNAs were found in HCC tumors. Among them, hsa_circ_001726 was highly expressed in HCC tumors and cells, which was transcribed from CCT2. As a transcription factor of CCT2, E2F6 knockdown inactivated CCT2 promoter and reduced hsa_circ_001726 expression. Moreover, hsa_circ_001726 elevated PRMT9 expression by sponging miR-671-5p, and then activated Notch signaling pathway. Additionally, hsa_circ_001726 deficiency repressed malignant phenotypes of HCC cells, including proliferation, migration, invasion, EMT and apoptosis. In vivo, hsa_circ_001726 deficiency reduced tumor growth and lung metastasis of HCC in xenograft mouse models and orthotopic transplantation tumor mouse models.

**Conclusion:**

Hsa_circ_001726 functioned as an oncogene in HCC, which was derived from CCT2 and regulated by E2F6. Hsa_circ_001726 elevated PRMT9 expression by sponging miR-671-5p, and then activated Notch signaling pathway, thereby accelerating malignant phenotypes of HCC. Therefore, targeting hsa_circ_001726 may be a new avenue for HCC treatment.

**Supplementary Information:**

The online version contains supplementary material available at 10.1186/s12885-023-11703-7.

## Introduction

Hepatocellular carcinoma (HCC) is one of the most common malignant tumors worldwide, characterized by poor prognosis and susceptibility to early metastasis [1]. Radical resection surgery or liver transplantation has become the main treatment method for HCC. For late stage patients who have lost the opportunity for surgery, they can only be forced to receive chemotherapy drugs as the main treatment method [2, 3]. But currently, HCC patients have a high risk of recurrence and metastasis after surgery, resulting in a lower 5-year survival rate and poor prognosis [4]. HCC is prone to extrahepatic metastasis, with the lung being the most common site of metastasis, accounting for 51% of all extrahepatic metastases [5, 6]. Thus, searching for more effective diagnostic and therapeutic biomarkers for HCC is particularly important for improving the prognosis and quality of life for HCC patients. In recent years, circRNA has received widespread attention as an emerging biomarker for cancer diagnosis and treatment.

CircRNAs are a kind of special non-coding RNA molecule with closed loop structure, which widely exists in eukaryotic cells [7]. CircRNAs interact with microRNAs (miRNAs) and competitively block the interaction between miRNAs and their downstream mRNA, which impacts the functional expression of downstream target mRNA [8]. It has been found that circRNAs are essential regulators of many pathological or physiological processes, including tumorigenesis [9]. For instance, circRNA_102231 is highly expressed in tumor tissues and plasma samples of gastric cancer, which accelerates proliferation and invasion of gastric cancer cells [10]. Given that circRNA-SORE is up-regulated in HCC tumors, circRNA-SORE silencing may be able to overcome sorafenib resistance [11]. Hsa_circ_001726 is a novel circRNA, which is transcribed from Chaperonin containing TCP1 subunit 2 (CCT2). CCT2 is an aggrephagy receptor for clearance of solid protein aggregates [12]. CCT2 functions as an oncogenic gene to participate in the progression of various cancers, such as gastric cancer, colorectal cancer and HCC [13–15]. However, the functional role of CCT2-derived hsa_circ_001726 in HCC has not been reported.

MicroRNAs (miRNAs) are a group of endogenous non-coding RNA that typically complement and bind to the 3' or 5' non-coding regions of the target mRNA [16]. Minor changes in miRNA expression can affect hundreds of target genes and significantly alter transcriptomics. Therefore, miRNA disrupts various disease regulatory networks [17, 18]. MiR-671-5p has been reported to play a crucial role in various cancers, such as HCC, ovarian cancer and breast cancer [19–21]. Bioinformatics analysis has revealed that there are binding sits between hsa_circ_001726 and miR-671-5p. However, hsa_circ_001726 whether can affect HCC development by targeting miR-671-5p has not been reported.

Bioinformatics analysis has revealed that protein arginine methyltransferases 9 (PRMT9) is a target mRNA of miR-671-5p. PRMT9 is a protein in the PRMT family, which is a type II arginine methyltransferase [22]. Our previous study has confirmed that PRMT9 serves as an oncogene that plays an important role in HCC invasion and metastasis through epithelial mesenchymal transition (EMT) process [23]. EMT is considered as a key step in tumor invasion and metastasis [24]. Tumor cells acquire strong invasion and metastasis ability through the EMT process, allowing them to migrate from the primary lesion to other tissues and organs [25]. This process is jointly regulated by multiple signaling pathways, such as TGF-β, WNT, and Notch signaling pathways [26, 27]. Thus, the precise mechanism of PRMT9 in accelerating metastasis of HCC still needs further investigation.

In this work, we identified a new circRNAs, hsa_circ_001726, which is transcribed from CCT2 and regulated by E2F transcription factor 6 (E2F6). In HCC cells, the deficiency of hsa_circ_001726 repressed migration and invasion of HCC cells in vitro, as well as reduced tumor growth and lung metastasis of HCC in vivo. Hsa_circ_001726 regulated EMT process by activating Notch signaling pathway via miR-671-5p/PRMT9 axis, thereby accelerating the malignant progression of HCC.

## Materials and methods

### Bioinformatics analysis

The gene expression profile in liver hepatocellular carcinoma (LIHC) were obtained from The Cancer Genome Atlas (TCGA). GSE121714 dataset was download from Gene expression omnibus (GEO) database. GSE121714 dataset contained the circRNA expression profile in non-tumor tissues (Normal group, 10 samples), tumor tissues of HCC patients with metastasis (Metastasis group, 10 samples) and HCC patients without metastasis (Primary group, 10 samples). The differentially expressed circRNAs (DEcircRNAs) between Normal group and Primary group, Primary group and Metastasis group were identified with same R package. The cut-off criteria was |log_2_fold change|> 1 and *P*-value < 0.05. Applying “pheatmap” and “ggplot2” R packages, heatmap and volcanos of the DEcircRNAs were obtained. The Venn diagram was employed to take intersection of DEcircRNAs. CircBase database was used to retrieve the source of hsa_circ_001726. The PROMO database predicted the transcription factors that regulate the CCT2 promoter.

### Participants

Tumor tissues were obtained from HCC patients with metastasis (40 samples) and HCC patients without metastasis (40 samples). Unpaired adjacent nontumor tissues (40 samples) were collected as control. HCC patients were received hepatectomy in the first affiliated hospital of Nanchang University. The patients did not receive any treatment before surgery. Using pathological section examination, these patients were diagnosed with HCC.

### Cell culture

Normal human hepatic cell line LO2 and liver cancer cell lines (Huh7, MHCC97H, Sk-Hep1, Bel7402) were purchased from the Type Culture Collection of Chinese Academy of Sciences (Shanghai, China). All cells were cultured in DMEM or RPMI1640 medium (Gibco, Grand Island, NY, USA) supplemented with 10% fetal bovine serum (Gibco) and 1% penicillin–streptomycin (Sangon-Biotech, Shanghai, China). The culture condition was 37 °C and 5% CO_2_. MHCC97H and Huh7 cells were treated with RNase R (Sangon-Biotech) at 37 °C for 30 min.

### Cell transfection

Small interfering RNA (siRNA) specifically targeting E2F6 (si-E2F6: 5’-GGG TAT TCT TGA CTT AAA CAA-3’; 5’-GAA AGC GGA GAG TGT ATG ACA-3’; 5’- GAA GGC CCT GAG GAA GAA GAA-3’) or hsa_circ_001726 (si-hsa_circ_001726: 5’-GGC AAC CTG GAG GCA ATT CAT-3’; 5’-GCA ACC TGG AGG CAA TTC ATA-3’; 5’-GCA GAT TCC TAT TTA GAT GAA-3’), short hairpin RNA (shRNA) specifically targeting hsa_circ_001726 (sh-hsa_circ_001726: 5’-GGC AAC CTG GAG GCA ATT CAT TCA AGA GAT GAA TTG CCT CCA GGT TGC C-3’; 5’-GGC AAC CTG GAG GCA ATT CAT CTC TTG AAT GAA TTG CCT CCA GGT TGC C-3’), miR-671-5p inhibitor (miR-671-5p-In: 5’-CUC CAG CCC CUC CAG GGC UUC CU-3’) were used to silence E2F6, hsa_circ_001726 or miR-671-5p in liver cancer cells. MiR-671-5p mimic (5’-TGC AGG CTA GTC AAC TTA GGC ACG TCA A-3’) and pcDNA3.1 carrying PRMT9 (PRMT9-OE) were applied to overexpress miR-671-5p or PRMT9 in liver cancer cells. The si-NC (5’-TAG CGC TGA GGC GTG CAA GCT GAT TCT TA-3’), sh-NC (5’-GAT CCC CTT CTC CGA ACG TGT CAC GTT TCA AGA GAA CGT GAC ACG TTC GGA GAA TTT TT-3’), mimic NC (5’-TAG CCC AGA CAC TGC AGC GTA GCG ATC CA-3’), inhibitor NC (In-NC: 5’-CAG UAC UUU UGU GUA GUA CAA-3’) and empty pcDNA3.1 vector (Vector) were served as control. The sh-hsa_circ_001726 and sh-NC were packaged into lentiviral particles, generating (LV-sh-hsa_circ_001726 and LV-sh-NC). These vectors were obtained from Genepharm (Shanghai, China). Huh7 and MHCC97H cells were transfected with vectors or oligonucleotides applying Lipofectamine™2000 reagent (Thermo Fisher Scientific, San Jose, CA, USA). Huh7 cells were infected with LV-sh-hsa_circ_001726 or LV-sh-NC applying polybrene (MedChemExpress, Monmouth Junction, NJ, USA).

### quantitative real-time PCR (qRT-PCR)

Applying TRIzol reagent (Thermo Fisher Scientific), total RNA was extracted from cells and tissues. Cytoplasmic and nuclear RNA purification kit (Norgen Biotek, Canada) was utilized to extract cytoplasmic and nuclear RNA from cells. For detection of the structure of CCT2 and hsa_circ_001726, total RNA was digested with RNase R (Sangon-Biotech) at 37 °C for 30 min. Total RNA was served as template to synthesize complementary DNA applying PrimeScript reverse transcriptase reagent kit or Mir-X miRNA First-Strand Synthesis Kit (TaKaRa, Beijing, China). Real‐time PCR was performed on StepOnePlus Real-Time PCR System (Applied Biosystems, Foster City, CA, USA) applying TB Green® Premix Ex Taq™ II or Mir-X miRNA qRT-PCR TB Green® Kit (TaKaRa). GAPDH, β-actin and U6 were served as loading control. The primer sequences (5’-3’) were listed as follow: CCT2: forward-TGC GGG CAC AAC ATT ATC CT and reverse-TGC GCA AGA ACA CAA AGA GC; E2F6: forward-TGC CTT CGC CAT GAA TCC TT and reverse-TCG GAC TCC CAG TTT CGT TG; hsa_circ_001726: forward-TGC GGG CAC AAC ATT ATC CT and reverse-AGG TTG CCA GAG CCT TTC AG; miR-617-5p: forward-ACA CTC CAG CTG GGA GGA AGC CCT GG and reverse-CTC AAC TGG TGT CGT GGA GTC GGC AAT; PRMT9: forward-TCC TCT CCC ATT TCA ACC TGG and reverse-TCC TCT CCC ATT TCA ACC TGG; E-cadherin: forward-CCT GGG ACT CCA CCT ACA GA and reverse-AGG AGT TGG GAA ATG TGA GC; N-cadherin: forward-CAA GAT GGG TCA ATG GAA ATA G and reverse-CTC AGG AAT ACG AGC CTT CAC; GAPDH: forward-GCA CCG TCA AGG CTG AGA AC and reverse-TGG TGA AGA CGC CAG TGG A; β-actin: forward-ATC GTG CGT GAC ATT AAG GAG AAG and reverse-AGG AAG GAA GGC TGG AAG AGT G; U6: forward-GCT TCG GCA GCA CAT ATA CTand reverse-GGT GCA GGG TCC GAG GTA T. Data were analyzed by 2^−ΔΔCt^ method.

### Actinomycin D assay

The stability of CCT2 and hsa_circ_001726 was measured using Actinomycin D (Sigma-Aldrich, St. Louis, MO, USA) assay. Briefly, Huh7 cells were treated with 5 µg/mL Actinomycin at 37 °C for 24 h. At 0, 4, 8, 12, 24 hours after Actinomycin D treatment, total RNA was isolated from cells. The expression of CCT2 and hsa_circ_001726 was examined by performing qRT-PCR assay.

### Immunohistochemistry (IHC) assay

The dissected tissues from HCC patients were paraffin-embedded and cut into Sects. (4 μm). Following deparaffinization and hydration, sections were subjected to heat-induced antigen retrieval. The sections were incubated with anti-E-cadherin (1: 100 dilution; Abcam, Cambridge, MA, USA) or anti-N-cadherin (1: 100 dilution; Abcam) at 4 °C overnight, and then stained with goat anti-rabbit HRP-conjugated IgG (1: 2000 dilution; Abcam) at room temperature for 1 h. The sections were stained with 3,3'-Diaminobenzidine and then counterstained with Mayer’s hematoxylin.

### Western blotting

Tissues and cells were treated with RIPA lysis buffer (Beyotime, Shanghai, China), and total proteins were extracted. The protein samples were separated by performing 10% SDS-PAGE protein electrophoresis, and then transferred to a PVDF membrane (Millipore, Billerica, MA, USA). The membranes were incubated primary antibodies at 4 °C overnight, including anti-E2F6 (1: 1000 dilution; Abcam), anti-E-cadherin (1: 1000 dilution; Abcam), anti-ZO-1 (1: 1000 dilution; Abcam), anti-N-cadherin (1: 5000 dilution; Abcam), anti-Vimentin (1: 500 dilution; Abcam), anti-PRMT9 (1: 500 dilution; Abcam), anti-Notch1 (1: 1000 dilution; Abcam), anti-Hes 1 (1: 1000 dilution; Abcam) at room temperature for 1 h. The membranes were stained with goat anti-rabbit HRP-conjugated IgG (1: 2000 dilution; Abcam) at room temperature for 1 h. β-actin (1: 2000 dilution; Abcam) was served as loading control. The immunoprecipitated bands were developed with ECL reagent and analyzed by ImageJ software.

### 5-Ethynyl-2’-Deoxyuridine (EdU) assay

BeyoClick™ EdU Cell Proliferation Kit with Alexa Fluor 488 (Beyotime) was utilized to assess cell proliferation. MHCC97H and Huh7 cells (5 × 10^5^) were cultured in 6-well plates overnight, and then incubated with 10 μM EdU for 2 h. Cells were fixed with 4% paraformaldehyde for 10 min and permeabilized with 0.3% Triton X-100 for 20 min. Cells were stained with Alexa Fluor 488 azide in darkness for 30 min. Nucleus were stained with DAPI in darkness for 10 min. Finally, the proliferative cells were observed under a fluorescence microscope (Nikon, Tokyo, Japan).

### Wound healing

MHCC97H and Huh7 cells (5 × 10^5^) were seeded into 6-well plates and cultured at 37 °C and 5% CO_2_ until 100% confluence. Applying a 200-μL pipette tip, an artificial and straight scratch was created. Then, cells were washed with PBS to remove the suspended cells and cultured in serum-free medium. At 0 and 24 hours after cell culture, wound closure images were captured.

### Transwell invasion assay

A 24-well Transwell plate chamber with 8 μm pore size (Corning Costar, Cambridge, MA, USA) were used to examine cell invasion ability. Briefly, MHCC97H and Huh7 cells (1 × 10^6^ cells/ml) were seeded into the upper chamber coated with Matrigel. The lower chamber was added to the culture medium. After 24 hours incubation, the cells on the upper chamber were wiped off with a cotton swab, fixed with methanol for 10 min and stained with 0.1% crystal violet. The invasive cells were observed under an optical microscope.

### TUNEL staining

Apoptosis of HCC cells was evaluated using One Step TUNEL Apoptosis Assay Kit (Beyotime). Following 24 hours of incubation, MHCC97H and Huh7 cells were subjected to fixation with 4% paraformaldehyde and permeabilization with 0.3% Triton X-100. Cells were stained with 50 μL TUNEL detection reagent at 37 °C in darkness for 2 h. Finally, apoptotic cells were observed under a fluorescence microscope.

### RNA-fluorescence in situ hybridization (FISH)

Huh7 cells (1 × 10^4^ cells/well) were cultured in 48-well plate and fixed in 4% paraformaldehyde. Cells were permeabilized with 0.5% Triton X-100 for 15 min at 4 °C. Cy3-labeled hsa_circ_001726 probe were designed and synthesized by GenePharma. Cells were incubated with digoxigenin-labeled hsa_circ_001726 probe at 55 °C for 4 h, and then incubated with HRP-conjugated anti-DIG antibody (Jackson, West Grove, PA, USA). DAPI was used to stain nuclei. The fluorescence was observed under a confocal laser scanning microscope (Olympus, Tokyo, Japan).

### Chromatin immunoprecipitation (ChIP) assay

Pierce Magnetic ChIP Kit (Thermo Fisher Scientific) was utilized to verify the interaction between E2F6 and CCT2 in Huh7 and MHCC97H cells following the protocol of manufacturer. Briefly, cells were cultured in serum-free medium overnight for serum starvation. Cells were treated with 1% formaldehyde for 10 min. Following cell lysis, the chromatin was sonicated to obtain DNA fragment with a size of 500–1000 bp. Cell lysate was incubated with anti-E2F6 (Abcam) or isotype anti-IgG (Abcam) at 4 °C overnight. Then, cell lysate was treated with Protein A/G Magnetic Beads to obtain chromatin-antibody complexes. DNA was eluted from the chromatin-antibody complexes, and then analyzed by qRT-PCR.

### Luciferase reporter assay

To evaluate the influence of E2F6 on the activity of CCT2 promoter, CCT2 promoter was cloned into pGL3-basic, generating the vector pGL3-basic-CCT2 promoter. HEK-293 T cells were transfected with pGL3-basic-CCT2 promoter combined with si-E2F6/si-NC. For detection of the interaction relationship among hsa_circ_001726, miR-671-5p and PRMT9, the wild type or mutant hsa_circ_001726/PRMT9 containing the predictive binding site of miR-671-5p was cloned into the pmirGLO Dual-Luciferase miRNA Target Expression Vector (Promega, Madison, WI, USA), generating the vectors pmirGLO-hsa_circ_001726-WT (5’-UCC UAU UUA GAU GAU GGC UUC CU-3’), pmirGLO-hsa_circ_001726-MUT (5’-UCC UAU UUA GAU GAU CCG AAG GU-3’), pmirGLO-PRMT9-WT (5’-UGG GAA ACA CUG AAA GGC UUC CA-3’) and pmirGLO-PRMT9-MUT (5’-UGG GAA ACA CUG AAA CCG AAG GA-3’). HEK-293 T cells were transfected with WT/MUT of pmirGLO vectors and miR-671-5p mimic or mimic NC. At 48 h post-transfection, the luciferase assay was performed using the Dual Luciferase Reporter Assay System (Promega).

### RNA immunoprecipitation (RIP)

Applying Magna RIP kit (Millipore, Billerica, MA, USA), RIP assay was performed to verify the relationship between hsa_circ_001726 and miR-671-5p. Huh7 cells were treated with RIPA Lysis Buffer, cell lysates were collected for further use. Protein A/G magnetic beads were incubated with anti-AGO2 (Abcam) at room temperature for 1 h. Then, cell lysates were incubated with antibody-conjugated magnetic beads on a rotator at 4 °C for 12 h. IgG antibody (Abcam) served as control. The co-precipitated RNAs were purified from the bead-antibody-RNA complex, and then used for qRT-PCR analysis of hsa_circ_001726 and miR-671-5p.

### Animals

Immunodeficient BABL/c male nude mice (4–5 weeks old) were purchased from Shanghai SLAC Laboratory Animal Co. Ltd. Mice were maintained specific pathogen-free conditions with constant temperature (22–24 °C) and humidity (40–60%). Mice take food and water freely. The position of the mouse cage was rotated once a week, and the measurements of the mice were carried out in a fixed order. During the study, all protocols were carried out by experienced researcher applying double-blind method. Animal experiments were performed according to the Laboratory Animals-Guidelines for Ethical Review of Animal Welfare (GB/T 35892–2018) and the ARRIVE guidelines.

### Xenograft mouse model

According to statistical requirements and the feasibility of experimental operations, mice were randomly divided into two groups (*n =* 6): LV-sh-NC group and LV-sh-hsa_circ_001726 group. Mice were subcutaneously injected with 200 μL of Huh7 cells following transfection of LV-sh-hsa_circ_001726 or LV-sh-NC. Tumor volume was monitored every week for 5 weeks. On the 25^th^ day of modeling, all mice were anesthetized with intraperitoneal injection of ketamine (100 mg/kg body weight) and then euthanized with cervical dislocation. Tumor tissues were separated, and tumor weight was detected. After one week of inoculation, mice with subcutaneous tumor formation observed with the naked eye were included in the experiment. The weight of tumor tissues should not exceed 10% of the mouse's body weight, and the average tumor diameter should not exceed 20 mm. If ulceration occurs and severe metastasis leads to infection or necrosis, the experiment should be stopped and the animal should be euthanized.

### Orthotopic transplantation tumor model

Orthotopic transplantation tumor model was constructed as previous study described [28]. When the xenograft tumors grew to about 1 cm^3^ in volume, the tumor tissues were removed from the mice in each group (LV-sh-hsa_circ_001726 and LV-sh-NC), and then cut into small pieces (1 mm^3^). Mice were randomly divided into two groups (*n =* 6): LV-sh-NC group and LV-sh-hsa_circ_001726 group. Following anesthetization with 1% ketamine (100 mg/kg), the clipped tumor tissues were transplanted into the subcapsular region of the left medial lobe of the liver of nude mice. The 8–0 nylon surgical sutures were used to suture the liver incision. When the liver tumors reached about 20 mm in length, mice were euthanized with ketamine combined with cervical dislocation. Lung tissues were collected from mice. The number of metastatic nodules in the lung tissues were counted under a dissecting microscope. If ulceration occurs and severe metastasis leads to infection or necrosis, the experiment should be stopped and the animal should be euthanized.

### Hematoxylin–eosin (HE) staining

Lung tissues were fixed with 4% paraformaldehyde and embedded with paraffin. The paraffin sections with 4 μm thick were subjected to deparaffinization and hydration. After that, the sections were stained with hematoxylin for 5 min and incubated with eosin for 30 s applying HE staining kit (Beyotime). Finally, the pathological changes of lung tissues were observed under an optical microscope.

### Statistical analysis

Each assay was performed for 3 times. Data were analyzed by SPSS 22.0 statistical software (IBM, Armonk, NY, USA) and expressed as mean ± standard deviation. Two-tailed Student’s t test and one-way ANOVA were used to analyze the statistical difference. Kaplan Meier plotter was used to analyze prognosis survival curve. Spearman analysis was used for correlation analysis. *P < *0.05 was considered as a significant difference.

## Results

### The expression and characteristics of hsa_circ_001726 in HCC

**Fig. 1:**
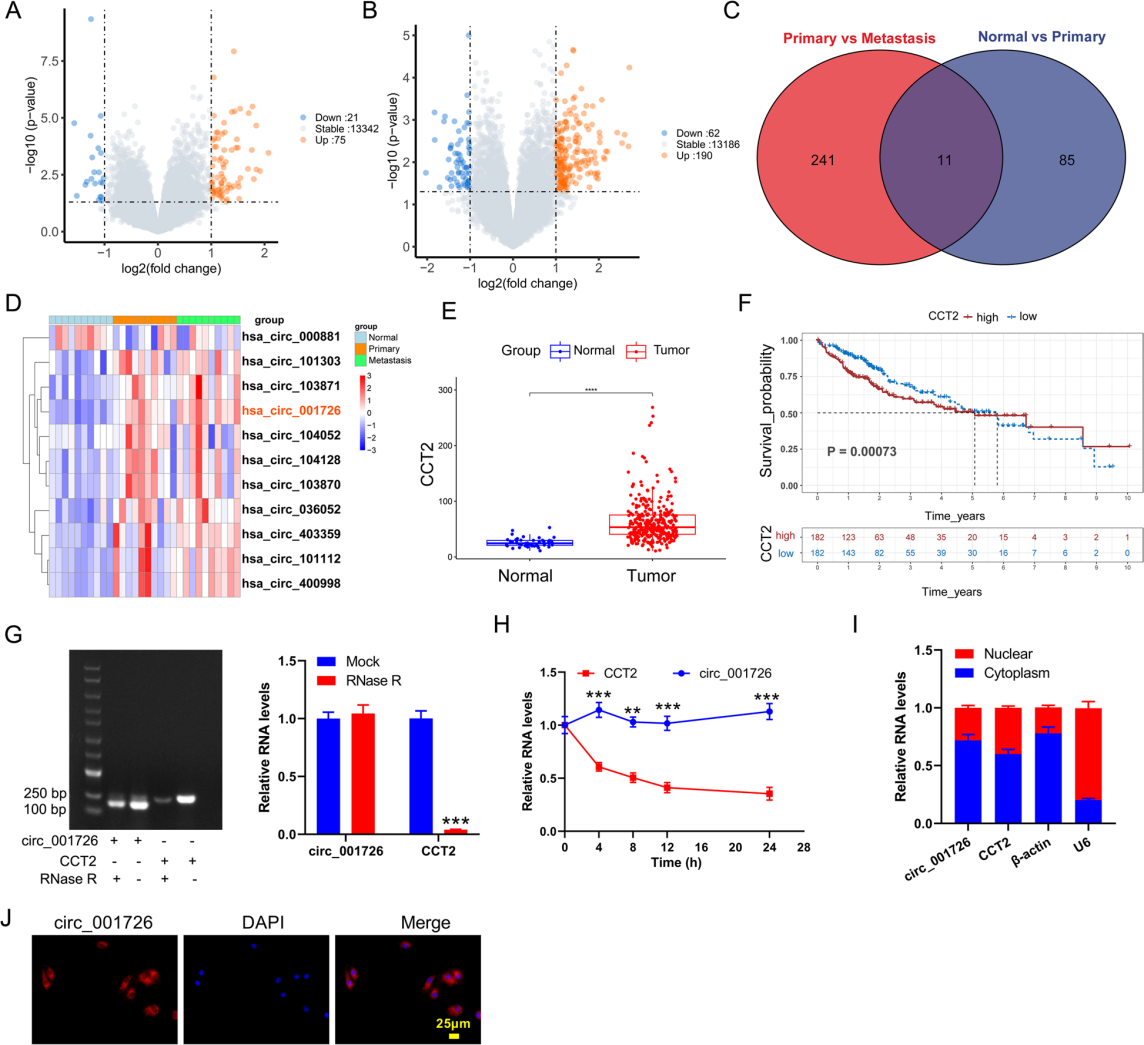
The expression and characteristics of hsa_circ_001726 in HCC. **A** Volcano Plot showing the differentially expressed circRNAs between adjacent nontumor tissues and tumor tissues without metastasis. **B** Volcano Plot showing the differentially expressed circRNAs between tumor tissues without metastasis and tumor tissues with metastasis. **C** The intersection of differentially expressed circRNAs. **D** Heatmap showing the 11 overlapping differentially expressed circRNAs. **E** The expression of CCT2 in tumor and normal tissues from TCGA-LIHC cohort. **F** Kaplan–Meier curves analyzed the connection between CCT2 expression and overall survival of HCC patients in TCGA-LIHC cohort. **G** QRT-PCR detected the expression of CCT2 and hsa_circ_001726 in Huh7 cells following RNase R treatment. **H** QRT-PCR examined the expression of CCT2 and hsa_circ_001726 in Huh7 cells following Actinomycin D treatment. **I** QRT-PCR assessed the expression of CCT2 and hsa_circ_001726 in nuclear and cytoplasm of Huh7 cells. **J** RNA-FISH assay identified the localization of hsa_circ_001726 in Huh7 cells. ^**^P < 0.01, ^***^P < 0.001 vs Mock, CCT2 group

To investigate the key circRNAs involved in metastasis of HCC, we analyzed the differentially expressed circRNAs among adjacent nontumor tissues and tumor tissues without metastasis based on GSE121714 dataset. Compared with adjacent nontumor tissues, 21 down-regulated circRNAs and 75 up-regulated circRNAs were found in HCC tumor tissues without metastasis (Fig. 1A). There were 62 down-regulated circRNAs and 190 up-regulated circRNAs in HCC tumor tissues with metastasis as compared to HCC tumor tissues without metastasis (Fig. 1B). Venn diagram and heatmap showed there were 11 overlapping differentially expressed circRNAs (Fig. 1C and D). Hsa_circ_001726 is a novel discovered differentially expressed RNA with the highest expression in HCC. Hsa_circ_001726 is located on chr12 (69,983,264–69,985,939) with a length of 304 bp. Like all circRNAs, hsa_circ_001726 exhibits a closed ring structure. It is not easily degraded by nucleic acid exonucleases and is more stable than linear RNA. In light of the fact that hsa_circ_001726 is transcribed from CCT2 (circBase database). Then, we examined the expression of CCT2 in HCC applying TCGA-LIHC database. As shown in Fig. 1E and F, CCT2 was highly expressed in tumor tissues, which indicated a poor prognosis. Then, the structure of hsa_circ_001726 and CCT2 was examined by qRT-PCR. Following RNase R digestion, hsa_circ_001726 could be amplified, but not linear CCT2 (Fig. 1G). Actinomycin D assay evaluated the stability of hsa_circ_001726 and CCT2, showing that hsa_circ_001726 was much more stable than CCT2 (Fig. 1H). Moreover, the distribution of hsa_circ_001726 and CCT2 in the nuclear and cytoplasm of Huh7 cells was detected by qRT-PCR. The expression of hsa_circ_001726 and CCT2 was abundant in the cytoplasm, but rarely in the nucleus (Fig. 1I). Consistently, RNA-FISH assay uncovered that hsa_circ_001726 was localized in the cytoplasm of Huh7 cells (Fig. 1J). Thus, these data showed that hsa_circ_001726 was transcribed from CCT2, and served as an oncogene in HCC.

### Hsa_circ_001726 silencing impeded malignant progression of HCC

**Fig. 2:**
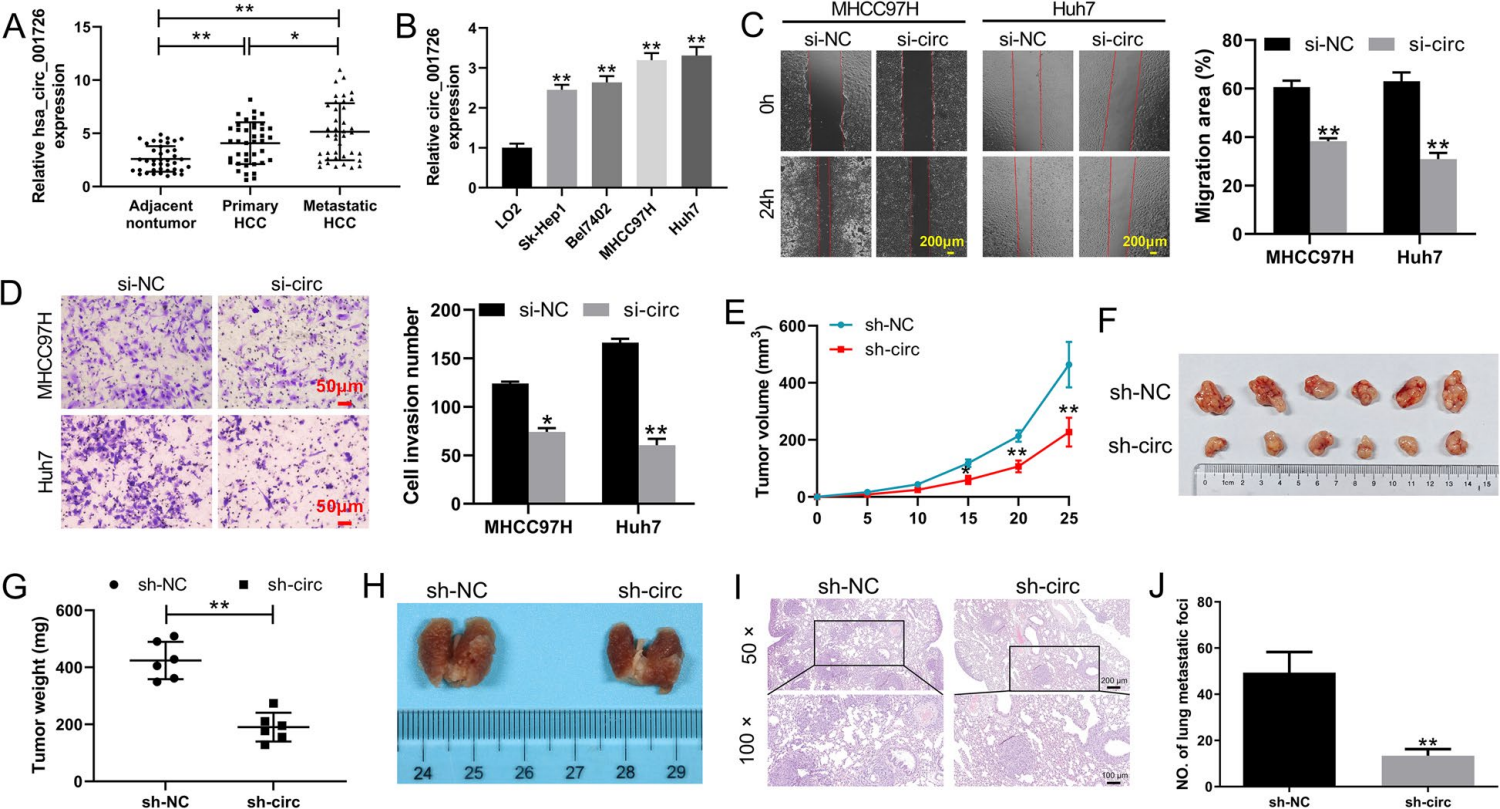
Hsa_circ_001726 deficiency inhibited HCC progression in vivo and in vitro. **A** QRT-PCR detected the expression of hsa_circ_001726 in adjacent nontumor tissues tumor tissues of HCC patients with or without metastasis. **B** QRT-PCR detected the expression of hsa_circ_001726 in LO2, Sk-Hep1, Bel7402, MHCC97H and Huh7 cells. **C**, **D** Wound healing and Transwell invasion assays examined migration and invasion of MHCC97H and Huh7 cells following transfection of si-hsa_circ_001726 or si-NC. Xenograft mouse model was constructed by inoculating Huh7 cells with LV-sh-hsa_circ_001726 or Huh7 cells with LV-sh-NC. **E**–**G** Tumor volume and weight of xenograft mice. Orthotopic transplantation tumor model was constructed by inoculating Huh7 cells with LV-sh-hsa_circ_001726 or Huh7 cells with LV-sh-NC. **H** The images of lung tissues. **I**, **J** HE staining examined the pathological changes of lung tissues. ^*^P < 0.05, ^**^P < 0.01 vs LO2, si-NC or sh-NC group

To understand the involvement of hsa_circ_001726 in HCC, the expression of hsa_circ_001726 in tumor tissues of HCC patients was examined by qRT-PCR. Compared with adjacent nontumor tissues, up-regulation of hsa_circ_001726 was observed in tumor tissues of primary HCC patients. The expression of hsa_circ_001726 was even higher in tumor tissues of metastatic HCC patients compared to those of tumor tissues of primary HCC patients (Fig. 2A). In addition, the precise mechanism of hsa_circ_001726 in HCC was determined in vitro. The results of qRT-PCR revealed that hsa_circ_001726 was up-regulated in HCC cell lines with respect to normal human hepatic cell line LO2 (Fig. 2B). Subsequently, we employed siRNA to silence hsa_circ_001726 and examined its impact on the malignant characteristics of HCC cells. Following transfection of si-hsa_circ_001726, the expression of hsa_circ_001726 was significantly decreased in MHCC97H and Huh7 cells (Supplementary Fig. 1A). Results obtained from wound healing and transwell assays showed that hsa_circ_001726 deficiency repressed migration and invasion of MHCC97H and Huh7 cells (Fig. 2C and D). Furthermore, a xenograft mouse model was constructed to verify the role of hsa_circ_001726 silencing in HCC in vivo. The expression of hsa_circ_001726 was severely decreased in Huh7 cells in the presence of LV-sh-hsa_circ_001726, as determined by qRT-PCR (Supplementary Fig. 1B). Hsa_circ_001726 knockdown led to a decrease in the volume and weight of tumor tissues in xenograft mice (Fig. 2E-G). Additionally, orthotopic transplantation tumor model was constructed to explore the influence of hsa_circ_001726 on tumor metastasis. Lung tissues are the most common site of extrahepatic metastasis in HCC [29]. The occurrence of lung metastasis is to some extent a manifestation of accelerated disease progression in patients, which increases their risk of death [6]. We observed the lung metastasis of mice. Hsa_circ_001726 deficiency obviously reduced the number of lung metastatic foci. Compared with LV-shNC group, orthotopic transplantation tumor mice with hsa_circ_001726 knockdown exhibited more serious lesions in lung tissues (Fig. 2H-J and Supplementary Fig. 2). Thus, these results collectively indicate that hsa_circ_001726 silencing impeded malignant progression of HCC in vivo and in vitro.

### Hsa_circ_001726 sponged miR-671-5p to elevate PRMT9 expression

**Fig. 3:**
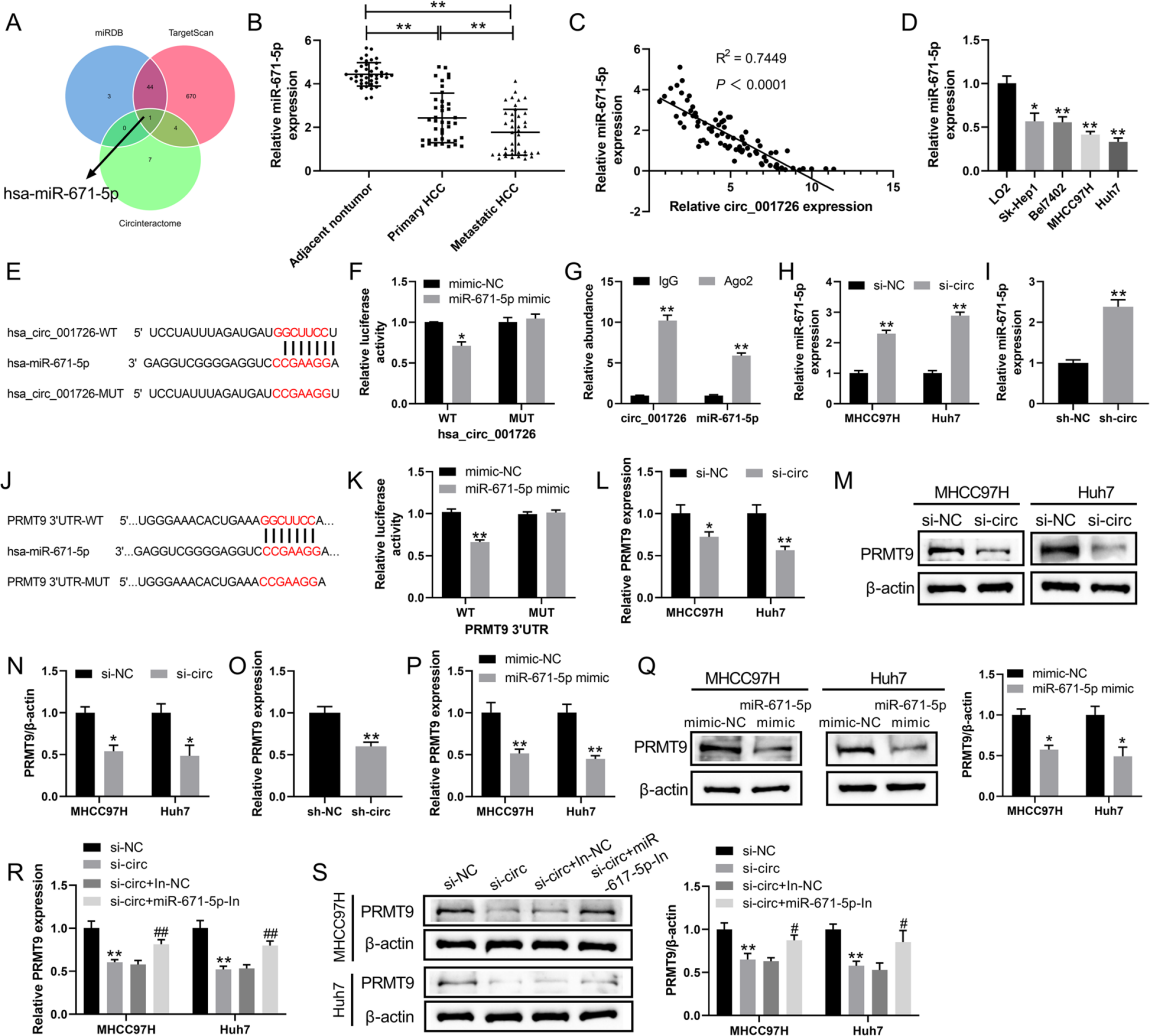
Hsa_circ_001726 sponged miR-671-5p to elevate PRMT9 expression. **A** The intersection of miRNAs from miRDB, TargetScan and Circinteractome. **B** QRT-PCR detected the expression of miR-671-5p in adjacent nontumor tissues, tumor tissues of HCC patients with or without metastasis. ^**^P < 0.01. **C** Person correlation analyzed the correlation between miR-671-5p and hsa_circ_001726. **D** QRT-PCR detected the expression of miR-671-5p in LO2, Sk-Hep1, Bel7402, MHCC97H and Huh7 cells. ^**^P < 0.01 vs LO2 group. **E**, **F** Luciferase reporter assay verified the interaction between miR-671-5p and hsa_circ_001726. ^**^P < 0.01 vs mimic-NC group. **G** RNA immunoprecipitation identified the interaction between miR-671-5p and hsa_circ_001726. ^**^P < 0.01 vs IgG group. **H** QRT-PCR assessed the expression of miR-671-5p in MHCC97H and Huh7 cells following transfection of si-hsa_circ_001726 or si-NC. ^**^P < 0.01 vs si-NC group. **I** QRT-PCR detected the expression of miR-671-5p in lung tissues of orthotopic transplantation tumor model with LV-sh-hsa_circ_001726 or LV-sh-NC. ^**^P < 0.01 vs sh-NC group. **J**, **K** Luciferase reporter assay verified the interaction between miR-671-5p and PRMT9. ^**^P < 0.01 vs mimic-NC group. **L**-**N** QRT-PCR and western blotting examined the expression of PRMT9 in MHCC97H and Huh7 cells following transfection of si-hsa_circ_001726 or si-NC. ^**^P < 0.01 vs si-NC group. ^**^P < 0.01 vs mimic-NC group. **O** QRT-PCR detected the expression of PRMT9 in lung tissues of orthotopic transplantation tumor model with LV-sh-hsa_circ_001726 or LV-sh-NC. ^**^P < 0.01 vs sh-NC group. **P**, **Q** QRT-PCR and western blotting examined the expression of PRMT9 in MHCC97H and Huh7 cells following transfection of miR-671-5p mimic or mimic NC. ^**^P < 0.01 vs mimic-NC group. **R**, **S** QRT-PCR and western blotting examined the expression of PRMT9 in MHCC97H and Huh7 cells following transfection of miR-671-5p-In or In-NC and si-hsa_circ_001726 or si-NC. ^**^P < 0.01 vs si-NC group; ^#^*P < *0.05, ^##^*P < *0.01 vs si-circ + In-NC group

Here, we analyzed the miRNA that targeted by hsa_circ_001726 based on circular RNA interactome database. Applying TargetScan and miRDB databases, the miRNA that interacted with PRMT9 was predicted. This analysis revealed an overlapping miRNA, miR-671-5p, which possesses binding sites in both hsa_circ_001726 and PRMT9 (Fig. 3A). Notably, miR-671-5p displayed significant downregulation in the tumor tissues of primary HCC patients compared to adjacent non-tumor tissues. The expression of miR-671-5p was lower in tumor tissues of metastatic HCC patients than that in primary HCC patients (Fig. 3B). The results of qRT-PCR exhibited a negative correlation between miR-671-5p expression and hsa_circ_001726 levels (Fig. 3C). Compared with normal human hepatic cell line LO2, miR-671-5p expression was also decreased in HCC cell lines (Fig. 3D). To verify the interaction between hsa_circ_001726 and miR-671-5p, luciferase reporter assay and RNA immunoprecipitation were carried out. The luciferase activity was notably decreased in HEK-293 T cells upon transfection with both miR-671-5p mimic and pmirGLO-hsa_circ_001726-WT, demonstrating a direct interaction between miR-671-5p and hsa_circ_001726 (Fig. 3E and F). AGO2 is an indicator protein for circRNA to exert sponge effects. MiRNA interacts with AGO2 protein to form RNA-induced silencing complex [30]. Anti-AGO2 increased the expression of hsa_circ_001726 and miR-671-5p as compared with anti-IgG (Fig. 3G), indicating that hsa_circ_001726 acted as sponge for miR-671-5p. Results of qRT-PCR showed that miR-671-5p was highly expressed in MHCC97H and Huh7 cells in the presence of si-hsa_circ_001726 (Fig. 3H). Similarly, miR-671-5p was up-regulated in lung tissues of orthotopic transplantation tumor mice with hsa_circ_001726 knockdown, as determined by qRT-PCR (Fig. 3I). Moreover, results of luciferase reporter assay showed that the luciferase activity was reduced in HEK-293 T following transfection of miR-671-5p mimic and pmirGLO-hsa_circ_001726-WT, indicating that miR-671-5p interacted with 3’ UTR of PRMT9 (Fig. 3J and K). Additionally, qRT-PCR and western blotting examined the impact of hsa_circ_001726 on the expression of PRMT9 in HCC cells. Hsa_circ_001726 silencing caused a down-regulation of PRMT9 in MHCC97H and Huh7 cells (Fig. 3L-N). In vivo, hsa_circ_001726 knockdown also reduced PRMT9 expression in lung tissues of orthotopic transplantation tumor mice (Fig. 3O). Then, miR-671-5p was overexpressed in MHCC97H and Huh7 cells applying transfection of miR-671-5p mimic (Supplementary Fig. 1C). MiR-671-5p mimic repressed PRMT9 expression in MHCC97H and Huh7 cells (Fig. 3P, Q). Furthermore, miR-671-5p was silenced in MHCC97H and Huh7 cells following transfection of miR-671-5p inhibitor (Supplementary Fig. 1D). QRT-PCR and western blotting results showed that hsa_circ_001726 knockdown repressed PRMT9 expression in MHCC97H and Huh7 cells, which was reversed by miR-671-5p inhibitor (Fig. 3R and S). All these data indicated that hsa_circ_001726 sponged miR-671-5p to elevate PRMT9 expression.

### Hsa_circ_001726 silencing repressed malignant phenotypes of HCC cells by regulating miR-671-5p/PRMT9 axis

**Fig. 4:**
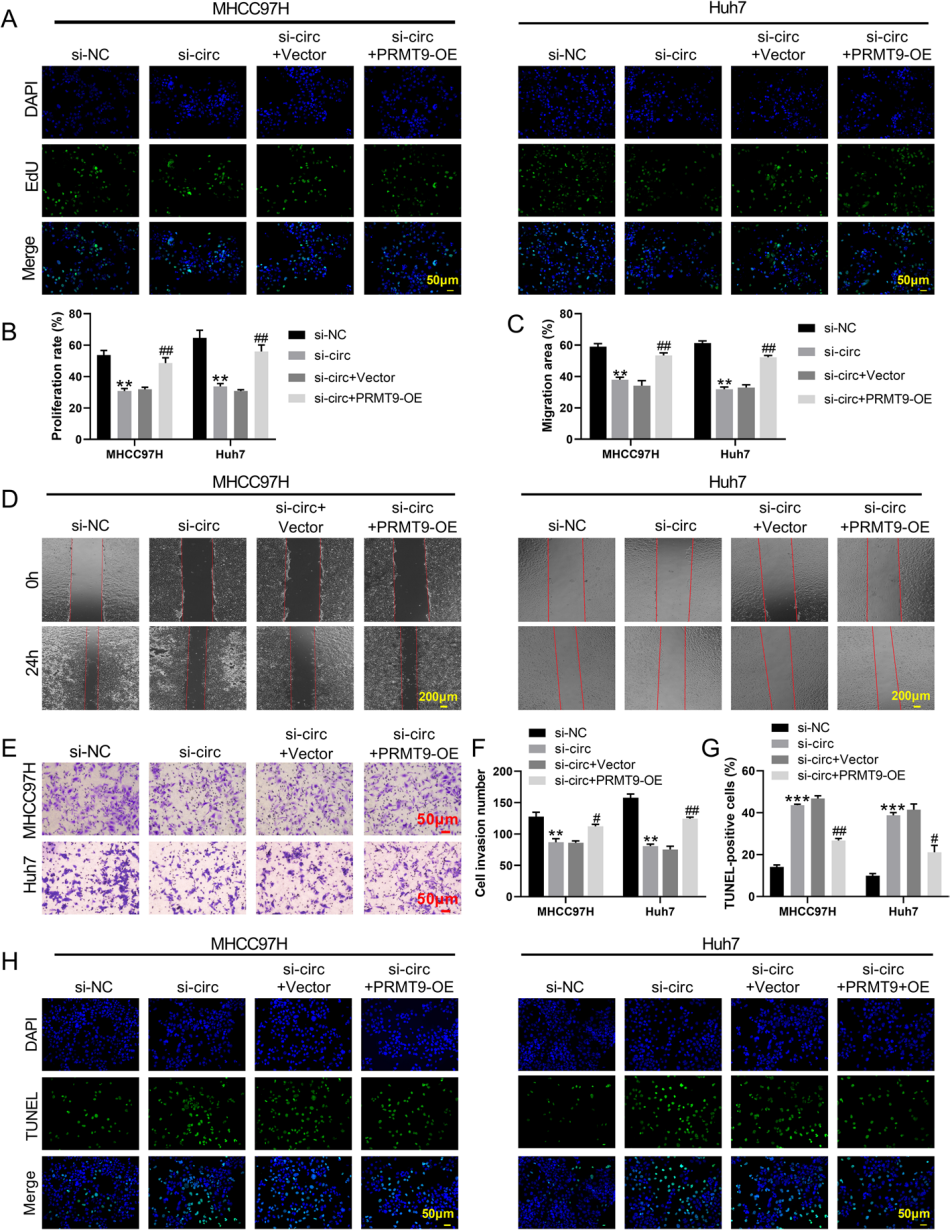
Hsa_circ_001726 silencing repressed proliferation, migration and invasion, and elevated apoptosis of MHCC97H and Huh7 cells by regulating PRMT9 expression. EdU staining (**A**, **B**), wound healing (**C**, **D**) and Transwell invasion (**E**, **F**) assays examined proliferation, migration and invasion of MHCC97H and Huh7 cells following transfection of hsa_circ_001726 or si-NC and PRMT9-OE or Vector. **G**, **H** TUNEL staining detected apoptosis of MHCC97H and Huh7 cells following transfection of hsa_circ_001726 or si-NC and PRMT9-OE or Vector. ^*^P < 0.05, ^**^P < 0.01 vs Vector or si-NC group; ^#^*P < *0.05, ^##^*P < *0.01 vs si-circ + Vector group

The mechanism of hsa_circ_001726/miR-671-5p/PRMT9 axis in HCC development was investigated. PRMT9 overexpression enhanced PRMT9 expression in MHCC97H and Huh7 cells, as determined by qRT-PCR and western blotting (Supplementary Fig. 1E, F). Then, the influence of hsa_circ_001726 on proliferation of HCC cells was detected by EdU staining. Hsa_circ_001726 silencing reduced proliferation of MHCC97H and Huh7 cells. Proliferation ability was elevated in MHCC97H and Huh7 cells in the presence of si-hsa_circ_001726 and PRMT9-OE (Fig. 4A and B). Wound healing and transwell invasion assays showed that hsa_circ_001726 silencing-mediated inhibition in migration and invasion of MHCC97H and Huh7 cells was rescued by PRMT9 overexpression (Fig. 4C-F). PRMT9 up-regulation reversed si-hsa_circ_001726-mediated promotion in apoptosis of MHCC97H and Huh7 cells, as determined by TUNEL staining (Fig. 4G and H). Moreover, miR-671-5p mimic severely repressed migration and invasion of MHCC97H and Huh7 cells (Supplementary Fig. 3A, B). Thus, hsa_circ_001726 silencing repressed malignant phenotypes of HCC cells, which may attribute to target regulate miR-671-5p/PRMT9 axis.

### Hsa_circ_001726 silencing repressed HCC development through Notch1/Hes 1-mediated EMT process

**Fig. 5:**
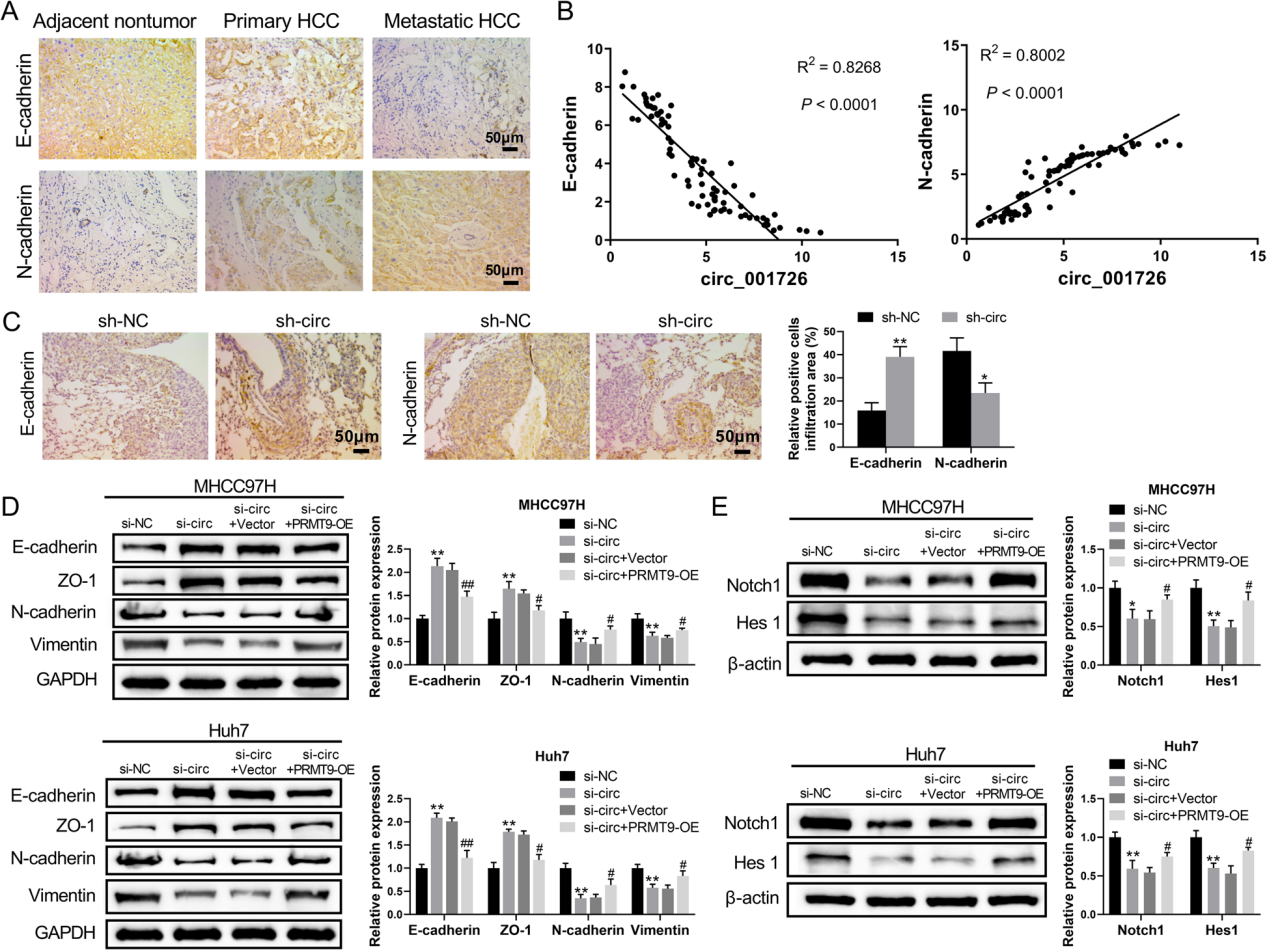
Hsa_circ_001726 knockdown inhibited HCC progression through Notch1/Hes 1-mediated EMT process. **A** IHC examined the expression of E-cadherin and N-cadherin in adjacent nontumor tissues, tumor tissues of HCC patients with or without metastasis. **B** Person correlation analyzed the correlation between hsa_circ_001726 and E-cadherin/N-cadherin in HCC. Orthotopic transplantation tumor model was constructed by inoculating Huh7 cells with hsa_circ_001726 deficiency. **C** IHC detected the expression of E-cadherin and N-cadherin in lung tissues. MHCC97H and Huh7 cells were transfected with hsa_circ_001726 or si-NC and PRMT9-OE or Vector. **D**, **E** Western blotting detected the expression of E-cadherin, ZO-1, N-cadherin, Vimentin, Notch1 and Hes 1 in MHCC97H and Huh7 cells. ^*^P < 0.05, ^**^P < 0.01 vs Vector, si-NC or sh-NC group; ^#^*P < *0.05, ^##^*P < *0.01 vs si-circ + Vector group. ^*^P < 0.05, ^**^P < 0.01 vs sh-NC group

EMT is an important mechanism of tumor metastasis [31]. Our previous study has confirmed that PRMT9 accelerates invasion and metastasis of HCC cells through EMT process [23]. We tested if hsa_circ_001726/miR-671-5p/PRMT9 affect HCC progression through EMT process. The expression of EMT markers in tumor tissues with high expression of hsa_circ_001726 was examined by immunohistochemistry (IHC) assay. Up-regulation of N-cadherin and down-regulation of E-cadherin were observed in primary and metastatic tumor tissues (Fig. 5A). The correlation between hsa_circ_001726 and N-cadherin/E-cadherin in HCC tumor tissues was analyzed. The results of qRT-PCR showed that hsa_circ_001726 expression was positively correlated with N-cadherin expression, and negatively correlated with E-cadherin expression in HCC tumor tissues (Fig. 5B). In an orthotopic transplantation tumor model, hsa_circ_001726 silencing led to an increase in E-cadherin expression and a decrease in N-cadherin expression in lung tissues, as determined by IHC assays (Fig. 5C). Moreover, the expression of EMT-related proteins in HCC cells was examined by western blotting analysis. Silencing of hsa_circ_001726 led to an up-regulation of E-cadherin and ZO-1, and a down-regulation of N-cadherin and Vimentin in MHCC97H and Huh7 cells, which was abolished by PRMT9 overexpression (Fig. 5D). Additionally, Notch1/Hes1 signaling pathway participates in EMT of cancer cells [32]. We also examined the Notch1/Hes1 signaling pathway, which is known to participate in the EMT of cancer cells. The expression of Notch1 and Hes1 was decreased in MHCC97H and Huh7 cells in the presence of si-hsa_circ_001726, while increased in MHCC97H and Huh7 cells following transfection of si-hsa_circ_001726 and PRMT9-OE (Fig. 5E). These results collectively indicated that hsa_circ_001726 silencing repressed HCC development through Notch1/Hes 1-mediated EMT process.

### Hsa_circ_001726 was regulated by E2F6 in HCC

**Fig. 6:**
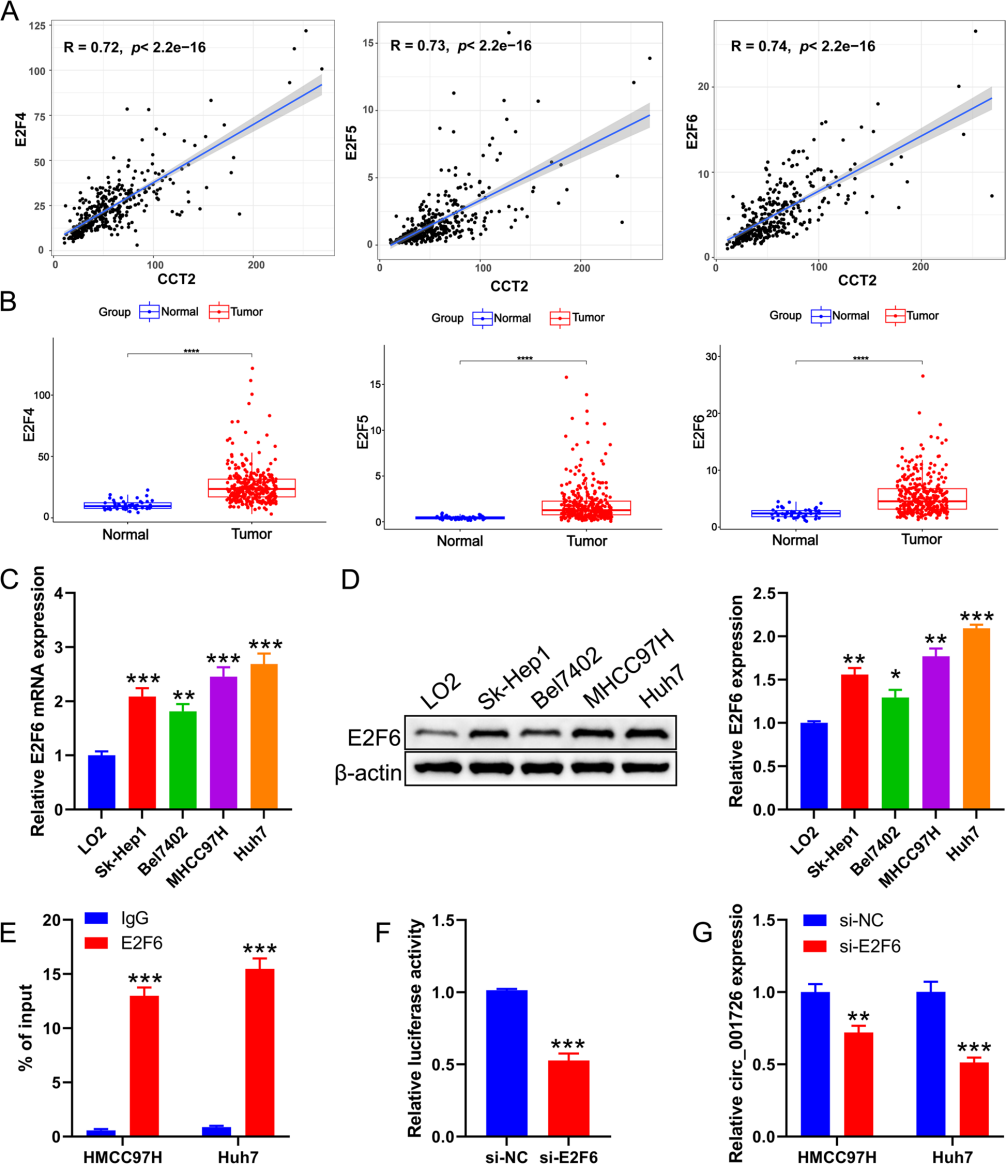
Hsa_circ_001726 was regulated by E2F6. **A** Correlations between CCT2 and E2F4/E2F5/E2F6 in TCGA-LIHC cohort was evaluated by Spearman analysis. **B** The expression of E2F4/E2F5/E2F6 in tumor and normal tissues from TCGA-LIHC cohort. **C**, **D** QRT-PCR and western blotting evaluated the expression of E2F6 in LO2, Huh7, MHCC97H, Sk-Hep1 and Bel7402 cells. **E** ChIP assay verified the interaction between E2F6 and CCT2 in Huh7 and MHCC97H cells. **F** Luciferase reporter assay examined the activity of CCT2 promoter. **G** QRT-PCR detected the expression of hsa_circ_001726 in Huh7 and MHCC97H cells following of si-E2F6 and si-NC. ^*^P < 0.05, ^**^P < 0.01, ^***^P < 0.001 vs LO2, IgG, si-NC group

The carcinogenic role of hsa_circ_001726 in HCC propelled us to further examine the underlying mechanism of its high expression. Hsa_circ_001726 is transcribed from CCT2. We speculated that the transcription factors involved in the transcription of CCT2 may have a similar effect on hsa_circ_001726. Applying PROMO database, we found there were 85 transcription factors may bind to the CCT2 promoter region. Among them, transcription factors with a correlation coefficient greater than 0.7 with CCT2, including E2F4, E2F5, and E2F6, especially E2F6 (Fig. 6A). Analysis of TCGA-LIHC database showed that E2F4, E2F5, and E2F6 were upregulated in tumor tissues of HCC compared to normal tissues (Fig. 6B). Furthermore, the expression of E2F6 in HCC cell lines was detected by qRT-PCR and western blotting, showing significant upregulation in Huh7, MHCC97H, Sk-Hep1, and Bel7402 cells compared to the normal hepatic cell line LO2 (Fig. 6C and D). These data indicated that E2F6 has a role in HCC development. To test if E2F6 can interact with CCT2 promoter, ChIP assay was carried out. Anti-E2F6 notably elevated the level of CCT2 in Huh7 and MHCC97H cells, indicating that E2F6 binds to the CCT2 promoter (Fig. 6E). Luciferase reporter assay revealed that the activity of luciferase was inhibited by E2F6 knockdown, showing that E2F6 knockdown repressed the activity of CCT2 promoter (Fig. 6F). Thus, we speculated that E2F6, as a transcription factor of CCT2, regulated the transcription of CCT2 and also affected the expression of hsa_circ_001726. E2F6 was silenced in Huh7 and MHCC97H cells, and the influence of E2F6 on hsa_circ_001726 expression was evaluated by qRT-PCR. E2F6 was severely down-regulated in Huh7 and MHCC97H cells in the presence of si-E2F6-2 and si-E2F6-3, especially si-E2F6-3 (Supplementary Fig. 1G, H). The expression of hsa_circ_001726 was notably decreased in Huh7 and MHCC97H cells upon transfection of si-E2F6 (Fig. 6G). Taken together, these findings suggested that hsa_circ_001726 was regulated by E2F6 in HCC.

## Discussion

**Fig. 7:**
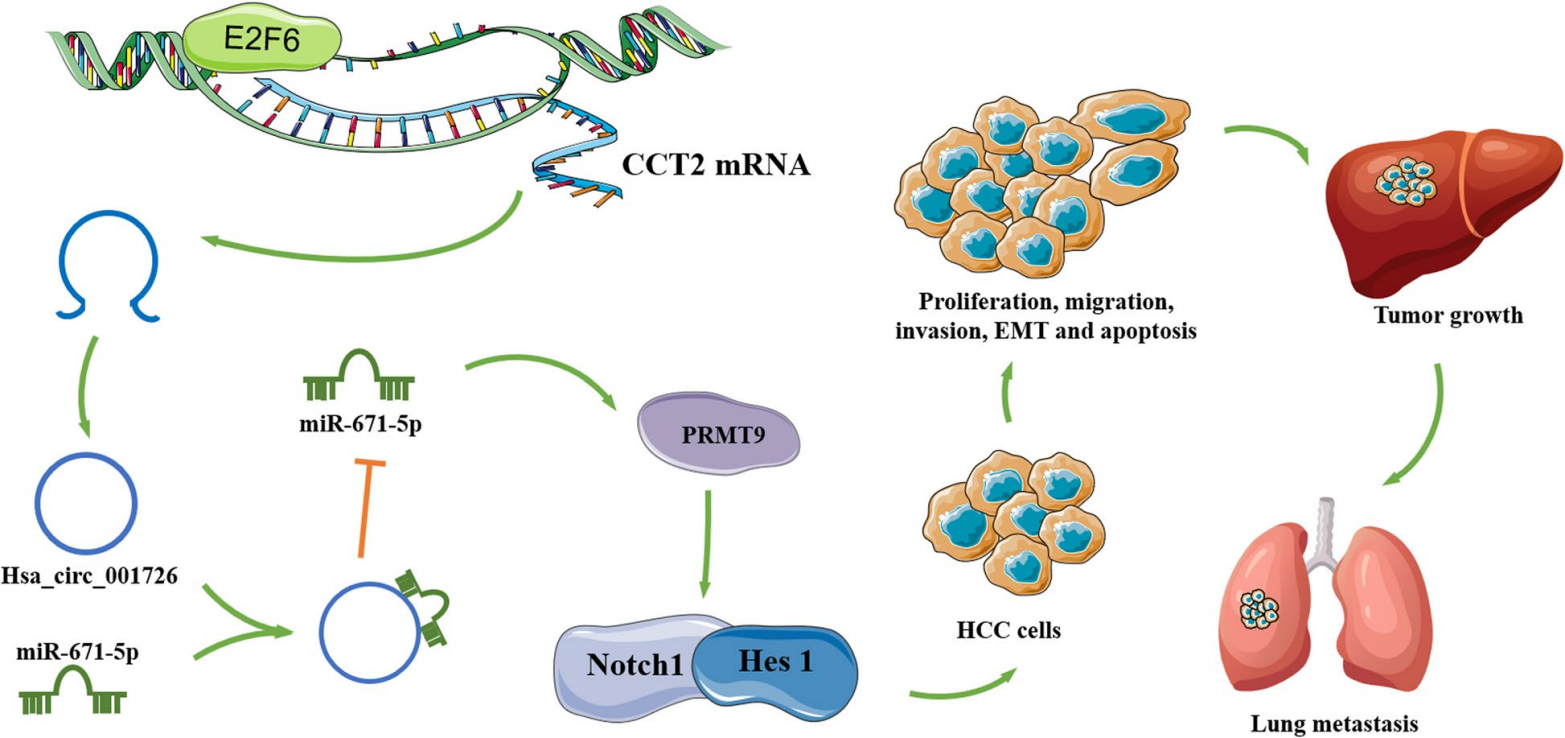
A schematic diagram of E2F6/hsa_circ_001726/miR-671-5p/PRMT9 axis in HCC. Hsa_circ_001726 was derived from CCT2 and regulated by E2F6. Hsa_circ_001726 elevated PRMT9 expression by sponging miR-671-5p, and then activated Notch signaling pathway, thereby accelerating proliferation, migration, invasion, apoptosis, EMT and metastasis of HCC

CircRNA is a class of non-coding RNAs with important regulatory potential, involved in the occurrence of various cancers. In this work, we found a novel circRNA, hsa_circ_001726, which is transcribed from CCT2. As a transcription factor of CCT2, E2F6 knockdown repressed the transcription of CCT2 and then reduced the expression of hsa_circ_001726. Up-regulation of hsa_circ_001726 was observed in tumor tissues of HCC patients with metastasis and HCC patients without metastasis. Higher expression of hsa_circ_001726 was closely associated with poor prognosis of HCC patients. Moreover, hsa_circ_001726 acted as a sponge for miR-671-5p, and then elevated the expression of PRMT9. Hsa_circ_001726 accelerated malignant phenotypes of HCC cells by regulating miR-671-5p/PRMT9 axis, including proliferation, migration, invasion, apoptosis and Notch1/Hes 1-mediated EMT. In vivo, hsa_circ_001726 deficiency reduced tumor growth and metastasis of HCC. Therefore,

hsa_circ_001726 elevated PRMT9 expression by sponging miR-671-5p, and then activated Notch signaling pathway to accelerate malignant phenotypes of HCC, as summarized in Fig. 7. Hsa_circ_001726 may be a biomarker for diagnosis and prognosis of HCC.

Bioinformatics is an emerging discipline that can analyze and interpret vast and complex biological information data, thereby revealing the biological mysteries hidden in genes. Applying bioinformatics analysis, human have recognized the vital role of circRNAs in various diseases, including many types of cancers [33]. Zhang et al. have applied bioinformatics analysis and screened abnormally expressed mRNAs, lncRNAs, and circRNAs in HCC tumor tissues, and constructed a prognostic circRNA-lncRNA-miRNA-mRNA ceRNA network containing 21 circRNAs, 15 lncRNAs, 5 miRNAs and 7 mRNAs [34]. Applying bioinformatics method, a novel up-regulated circRHOT1 is selected, which contributes to repress HCC progression via recruiting TIP60 to initiate NR2F6 expression [35]. In this work, we also carried out bioinformatics analysis of the circRNA expression profile in HCC tumor tissues based on GSE121714 dataset. It was found that there were 11 up-regulated circRNAs in tumor tissues of HCC patients without metastasis. Compared with tumor tissues without metastasis, these 11 circRNAs were further up-regulated in tumor tissues of HCC patients with metastasis. CircRNAs have several significant characteristics, including stability, universality, specificity, and conservation. These characteristics make them potential biomarkers for human diseases [36]. Thus, these 11 circRNAs may become potential biomarkers for HCC.

In the present work, we investigated the functional role of hsa_circ_001726 in HCC was studied for the first time. Hsa_circ_001726 is derived from the parental gene CCT2, which has previously been reported to be highly expressed in HCC tumor tissues, correlating with unfavorable clinical outcomes for HCC patients [15, 37]. Consistently, hsa_circ_001726 was highly expression in tumor tissues of HCC, where it played a significant role in promoting cancer. CircRNAs, like hsa_circ_001726, are known to be more resistant to exonucleases and represent promising candidates for RNA-based therapy. As a result, hsa_circ_001726 may have a more prominent role as a therapeutic target in cancer treatment compared to its parental gene CCT2. Given the crucial role of hsa_circ_001726 in cancer, we further explored the underlying mechanism of high expression of hsa_circ_001726 in HCC. It was found that transcription factor E2F6 activates the activity of CCT2 promoter, and then elevated the transcription of hsa_circ_001726. TCGA-LIHC database showed that E2F6 expression was increased in HCC tumors. Previous studies also confirmed the carcinogenic effect of E2F6 in HCC [38, 39]. Thus, hsa_circ_001726 functioned as an oncogene in HCC, which was regulated by E2F6.

Hsa_circ_001726 expression was found to be higher in HCC patients without metastasis, and further elevated in HCC patients with metastasis. Up-regulation of hsa_circ_001726 in HCC tumor tissues was positively correlated with N-cadherin expression, and negatively correlated with E-cadherin expression. The formation of circRNAs is mediated by EMT process, but the specific mechanism is unclear. Upregulation of N-cadherin and downregulation of E-cadherin are hallmark of EMT process, which is known to drive tumorigenesis and metastasis in cancers [40, 41]. Thus, hsa_circ_001726 may accelerate metastasis of HCC by promoting EMT process, and serve as a biomarker for poor prognosis of HCC patients. Moreover, in vitro results demonstrated that hsa_circ_001726 knockdown led to the suppression of proliferation, migration, invasion, apoptosis and EMT of HCC cells. Notch signaling pathway-related proteins Notch1 and Hes 1 were down-regulated in HCC cells by hsa_circ_001726 deficiency. Notch signaling pathway is closely associated with EMT process [42]. Our results align with previous studies showing the role of Notch in EMT regulation [43]. Consequently, hsa_circ_001726 knockdown repressed HCC development by inactivating Notch/Hes 1 signaling-mediated EMT process. Additionally, the results of in vivo assay showed that hsa_circ_001726 silencing inhibited tumor growth of HCC in xenograft mouse model, and suppressed lung metastasis of HCC in orthotopic transplantation tumor mouse model. Therefore, hsa_circ_001726 may be a potential target for HCC treatment, particularly in cases with lung metastasis.

Numerous researches have shown that circRNAs can be served as potential endogenous competitive RNA (ceRNA) molecules by interacting with miRNAs [44, 45]. Dysregulated miRNA expression has been found to be associated with various human tumors [46]. This work validated the ceRNA mechanism involving hsa_circ_001726, miR-671-5p and PRMT9. Hsa_circ_001726 elevated PRMT9 expression by sponging miR-671-5p. Moreover, miR-671-5p was identified as a tumor suppressor gene, inhibiting HCC cell migration, invasion, and EMT. A previous study has also confirmed the anti-tumor effect of miR-671-5p in HCC. Under hypoxic microenvironment, miR-671-5p silencing elevates TUFT1 expression to promote growth, EMT and metastasis of HCC cells [19]. However, Chen et al. have found that miR-671-5p functions as an oncogenic factor to accelerate malignant phenotypes of HCC cells by targeting ALDH2 [47]. Thus, the precise role of miR-671-5p in HCC is very complex, and needs further investigation.

PRMT9 is a type II arginine methyltransferase that catalyzes the monomethylation or symmetrical dimethylation of arginine residues in target proteins [22]. A case report has demonstrated that the homozygous PRMT9 (c.G40T:p.G14C) mutations is closely associated with the incidence of lung adenocarcinoma [48]. PRMT9 is decreased in the prostate tumor tissues as compared with non-neoplastic prostate tissues [49]. Our previous study has substantiated the role of PRMT9 as an oncogene in HCC. High expression of PRMT9 is associated with poor prognosis of HCC patients [23]. In the present study, we conducted an in-depth research on the mechanism of action of PRMT9 in HCC. We found that PRMT9 was regulated by hsa_circ_001726/miR-671-5p axis, and this regulation has significant implications for HCC growth and metastasis. The above studies indicate that the role of PRMT9 appears to vary among different cancers.

Despite these significant findings, there are limitations to our study. For instance, we screened 11 differentially expressed circRNAs in HCC. Among them, the precise mechanism of hsa_circ_001726 in HCC was determined. However, the underlying mechanisms of other genes in HCC are still unclear and require further research. Additionally, as a type II methyltransferase, PRMT9 may regulate methylated modification of target proteins, and then affect HCC development. The complex and context-dependent role of PRMT9 in HCC warrants further investigation.

## Conclusion

In summary, this work found that hsa_circ_001726 functioned as an oncogene in HCC, which was derived from CCT2 and regulated by E2F6. Hsa_circ_001726 elevated PRMT9 expression by sponging miR-671-5p, and then activated Notch signaling pathway, thereby accelerating proliferation, migration, invasion, apoptosis, EMT and metastasis of HCC. Therefore, targeting hsa_circ_001726 may be a new avenue for HCC treatment.

### Supplementary Information

**Additional file 1:Supplementary Fig. 1. **Detection of transcription efficiency. (A) QRT-PCR assessed the expression of hsa_circ_001726 in MHCC97H and Huh7 cells following transfection of si-hsa_circ_001726 or si-NC. (B) QRT-PCR detected the expression of hsa_circ_001726 in Huh7 cells following transfection of LV-sh-hsa_circ_001726 or LV-sh-NC. (C) QRT-PCR assessed the expression of miR-671-5p in MHCC97H and Huh7 cells following transfection of miR-671-5p mimic or mimic NC. (D) QRT-PCR examined the expression of miR-671-5p in MHCC97H and Huh7 cells following transfection of miR-671-5p-In or In-NC. (E-F) QRT-PCR and western blotting examined the expression of PRMT9 in MHCC97H and Huh7 cells following transfection of PRMT9-OE or Vector. (G-H) QRT-PCR and western blotting assessed the expression of E2F6 in Huh7 and MHCC97H cells following of si-E2F6 and si-NC. ^*^P < 0.05, ^**^P < 0.01 vs si-NC, shNC, mimic-NC, In-NC, Vector group. **Supplementary Fig. 2. **Hsa_circ_001726 knockdown alleviated lung metastasis in orthotopic transplantation tumor model. Orthotopic transplantation tumor model was constructed by inoculating Huh7 cells with LV-sh-hsa_circ_001726 or Huh7 cells with LV-sh-NC. HE staining examined the pathological changes of lung tissues. The full scan of HE staining. **Supplementary Fig. 3. **MiR-671-5p overexpression reduced migration and invasion of MHCC97H and Huh7 cells. (A-B) Wound healing and Transwell invasion assays examined migration and invasion of MHCC97H and Huh7 cells following transfection of miR-671-5p mimic or mimic NC. ^**^P < 0.01 vs mimic-NC group.

## Data Availability

The data that support the findings of this study are available from the corresponding author upon reasonable request.
