# Supplementary figures and images for: A hsa_circ_001726 axis regulated by E2F6 contributes to metastasis of hepatocellular carcinoma
Source: BMC Cancer. 2024 Jan 2;24:14. doi: 10.1186/s12885-023-11703-7 (PMC10763683; doi:10.1186/s12885-023-11703-7)

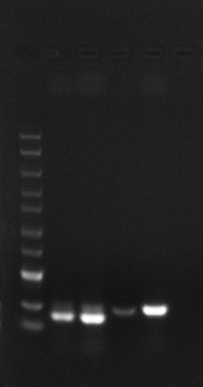

Supplement: Supplementary file 1 — Additional file 1:Supplementary Fig. 1. Detection of transcription efficiency. (A) QRT-PCR assessed the expression of hsa_circ_001726 in MHCC97H and Huh7 cells following transfection of si-hsa_circ_001726 or si-NC. (B) QRT-PCR detected the expression of hsa_circ_001726 in Huh7 cells following transfection of LV-sh-hsa_circ_001726 or LV-sh-NC. (C) QRT-PCR assessed the expression of miR-671-5p in MHCC97H and Huh7 cells following transfection of miR-671-5p mimic or mimic NC. (D) QRT-PCR examined the expression of miR-671-5p in MHCC97H and Huh7 cells following transfection of miR-671-5p-In or In-NC. (E-F) QRT-PCR and western blotting examined the expression of PRMT9 in MHCC97H and Huh7 cells following transfection of PRMT9-OE or Vector. (G-H) QRT-PCR and western blotting assessed the expression of E2F6 in Huh7 and MHCC97H cells following of si-E2F6 and si-NC. *P < 0.05, **P < 0.01 vs si-NC, shNC, mimic-NC, In-NC, Vector group. Supplementary Fig. 2. Hsa_circ_001726 knockdown alleviated lung metastasis in orthotopic transplantation tumor model. Orthotopic transplantation tumor model was constructed by inoculating Huh7 cells with LV-sh-hsa_circ_001726 or Huh7 cells with LV-sh-NC. HE staining examined the pathological changes of lung tissues. The full scan of HE staining. Supplementary Fig. 3. MiR-671-5p overexpression reduced migration and invasion of MHCC97H and Huh7 cells. (A-B) Wound healing and Transwell invasion assays examined migration and invasion of MHCC97H and Huh7 cells following transfection of miR-671-5p mimic or mimic NC. **P < 0.01 vs mimic-NC group. [file 12885_2023_11703_MOESM1_ESM.zip › DNA gel image of Figure 1G.tif]

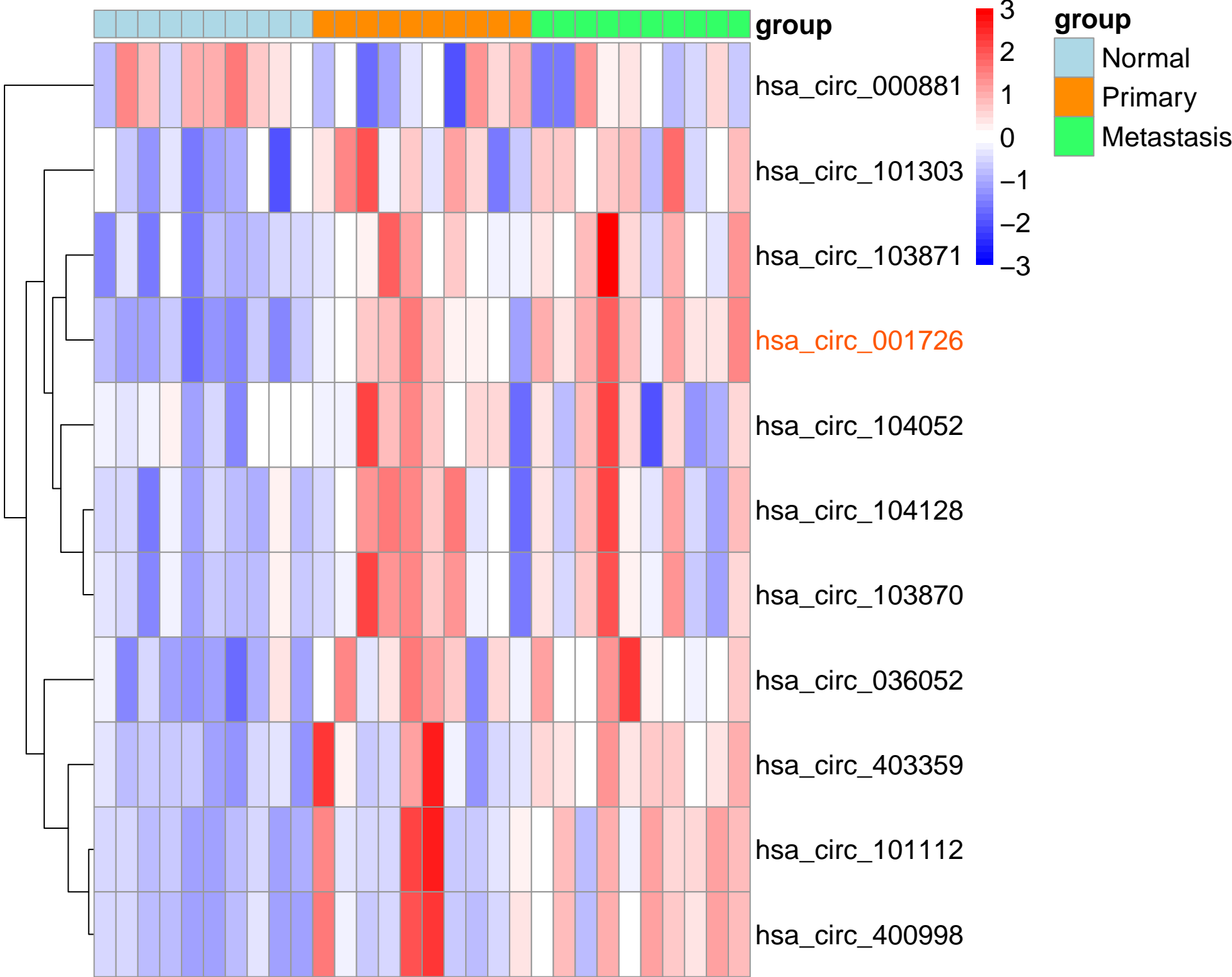

Supplement: Supplementary file 1 — Additional file 1:Supplementary Fig. 1. Detection of transcription efficiency. (A) QRT-PCR assessed the expression of hsa_circ_001726 in MHCC97H and Huh7 cells following transfection of si-hsa_circ_001726 or si-NC. (B) QRT-PCR detected the expression of hsa_circ_001726 in Huh7 cells following transfection of LV-sh-hsa_circ_001726 or LV-sh-NC. (C) QRT-PCR assessed the expression of miR-671-5p in MHCC97H and Huh7 cells following transfection of miR-671-5p mimic or mimic NC. (D) QRT-PCR examined the expression of miR-671-5p in MHCC97H and Huh7 cells following transfection of miR-671-5p-In or In-NC. (E-F) QRT-PCR and western blotting examined the expression of PRMT9 in MHCC97H and Huh7 cells following transfection of PRMT9-OE or Vector. (G-H) QRT-PCR and western blotting assessed the expression of E2F6 in Huh7 and MHCC97H cells following of si-E2F6 and si-NC. *P < 0.05, **P < 0.01 vs si-NC, shNC, mimic-NC, In-NC, Vector group. Supplementary Fig. 2. Hsa_circ_001726 knockdown alleviated lung metastasis in orthotopic transplantation tumor model. Orthotopic transplantation tumor model was constructed by inoculating Huh7 cells with LV-sh-hsa_circ_001726 or Huh7 cells with LV-sh-NC. HE staining examined the pathological changes of lung tissues. The full scan of HE staining. Supplementary Fig. 3. MiR-671-5p overexpression reduced migration and invasion of MHCC97H and Huh7 cells. (A-B) Wound healing and Transwell invasion assays examined migration and invasion of MHCC97H and Huh7 cells following transfection of miR-671-5p mimic or mimic NC. **P < 0.01 vs mimic-NC group. [file 12885_2023_11703_MOESM1_ESM.zip › Heatmap of Figure 1D.pdf]
